# Therapeutic Notes

**Published:** 1896-09-05

**Authors:** 


### Therapeutic Notes.

### THE THERAPEUTIC ACTION OF SALICYLIC ACID.

Although salicylic acid is one of the most extensively used drugs, its therapeutic action is but slightly known, and for the most part our knowledge is empyrical, and based on little accurate experimentation. A paper by M. G. Pouchet, in "Les Nouveaux Remèdes," February, 1896, dealing exhaustively with its chemical and therapeutic action, will be of interest to all who have occasion to prescribe this drug. The author, who regards salicylic acid in general terms as an antiseptic, that is to say, as a body which diminishes the physico-chemical function of cells, strongly insists that it is primarily and chiefly a poison of the cells of the nervous system, central and peripheral, a selectiveness in its action which he explains as due to the excessive vascularity, active nutrition and vitality of the protoplasm, of which they consist. Under such conditions the carbonic acid tension is high, and for the liberation of salicylic acid from salicylate of soda, it can be shown chemically that a high tension of carbonic acid is necessary. Although Vulpian, and others with him, believe that salicylate of soda has a specific action on cell activity, most authorities, including Pouchet, insist that it is only when the salicylic acid is freed from its combination that it has the therapeutic properties on which its value depends. Salicylic acid is not usually regarded as having a special influence on nerve cells or nerve structures, but Pouchet maintains that voluntary, reflex, and so-called automatic action, as well as sensation, are all affected by the drug, although the degree of sensitiveness to its influence is different in the different cases. Death in animals, and in one case in man, showed that the fatal effect was due to nerve paralysis. With regard to its specific action in cases of acute rheumatism and gout, it has been thought by many that an influence so marked must be due to an inhibition of septic processes due to its antiseptic properties. Pouchet, however, believes that its effect is due to other causes. As Ewald has shown, in rheumatism the carbonic acid tension in the inflamed areas may rise to three times its normal degree, and hence in these parts the conditions are present for the liberation of salicylic acid from its combinations. The acid set free restrains the protoplasmic action of the cells of the synovial and perisynovial tissues, limits their powers of secretion, lowers the fluid tension, allows the absorption, and thus relieves the pain. Laboratory experiments confirm this view as to the liberation of salicylic acid—for in animals which have been killed subsequent to the administration of salicylate of soda there has been found in the tissues of the articulations considerable quantities of free acid. In chronic inflammation, rheumatoid arthritis for instance, the necessary conditions, namely, high tension of carbonic acid, are not present in the diseased areas, and hence the inefficacy of the salicylic treatment in such conditions. The ordinary symptoms caused by salicylates are undoubtedly due to their selectiveness for nerve cells; the increased glandular activity, salivary, liver, &c., is due to central nervous causes, and the same is true of the heart and vascular system, as can be shown by experimentation on animals. The chief lessons that are to be learnt from Pouchet's observations are: (1) that salicylic acid must be regarded as specially affecting the central nervous system; (2) that salicylate of soda is only decomposed where protoplasmic activity is excessive, and where the carbonic acid tension is high; (3) that it is a drug for symptoms and not for diatheses, and should be employed only as a temporary expedient—as opposed to quinine in malaria, and mercury in syphilis; it is no prophylactic against subsequent attacks; (4) that owing to its irritative qualities, salicylic acid should be given well diluted.

